# Inference of regulatory networks through temporally sparse data

**DOI:** 10.3389/fcteg.2022.1017256

**Published:** 2022-12-13

**Authors:** Mohammad Alali, Mahdi Imani

**Affiliations:** Department of Electrical and Computer Engineering, Northeastern University, Boston, MA, United States

**Keywords:** topology inference, maximum likelihood estimation, gene regulatory networks, Boolean dynamical systems, Bayesian optimization

## Abstract

A major goal in genomics is to properly capture the complex dynamical behaviors of gene regulatory networks (GRNs). This includes inferring the complex interactions between genes, which can be used for a wide range of genomics analyses, including diagnosis or prognosis of diseases and finding effective treatments for chronic diseases such as cancer. Boolean networks have emerged as a successful class of models for capturing the behavior of GRNs. In most practical settings, inference of GRNs should be achieved through limited and temporally sparse genomics data. A large number of genes in GRNs leads to a large possible topology candidate space, which often cannot be exhaustively searched due to the limitation in computational resources. This paper develops a scalable and efficient topology inference for GRNs using Bayesian optimization and kernel-based methods. Rather than an exhaustive search over possible topologies, the proposed method constructs a Gaussian Process (GP) with a topology-inspired kernel function to account for correlation in the likelihood function. Then, using the posterior distribution of the GP model, the Bayesian optimization efficiently searches for the topology with the highest likelihood value by optimally balancing between exploration and exploitation. The performance of the proposed method is demonstrated through comprehensive numerical experiments using a well-known mammalian cell-cycle network.

## 1 Introduction

Gene regulatory networks (GRNs) play an important role in the molecular mechanism of underlying biological processes, such as stress response, DNA repair, and other mechanisms involved in complex diseases such as cancer. The topology inference of GRNs is critical in systems biology since it can generate valuable hypotheses to promote further biological research. Furthermore, a deep understanding of these biological processes is key in diagnosing and treating many chronic diseases. Advances in high-throughput genomic and proteomic profiling technologies have provided novel platforms for studying genomics. Meanwhile, single-cell gene-expression measurements allow capturing multiple snapshots of these complex biological processes. These advances offer an opportunity for seeking systematic approaches to understand the structure of GRNs.

In recent years, Boolean network models have been successfully employed for modeling different biological networks (Wynn et al., 2012; Saadatpour and Albert, 2013; Abou-Jaoudé et al., 2016). More specifically, these Boolean networks have been widely used for inferring GRNs from their state (i.e., gene) data (Pusnik et al., 2022). The state value of genes in the Boolean network is represented by 1 and 0, representing the activation and inactivation of genes, respectively. There are several Boolean network models, including the deterministic Boolean network models, Boolean network with perturbation, probabilistic Boolean network models, and Boolean control networks (Lähdesmäki et al., 2003; Shmulevich and Dougherty, 2010; Cheng and Zhao, 2011). Most of these models account for genes’ stochasticity and can effectively capture the dynamics of GRNs through relatively small times-series data.

Inference of Boolean network models consists of learning the parameters of their models given all the available data. Several advances have been made in the inference of Boolean network models in recent years. These techniques aim to find models that best fit the available time-series data. The fitness criteria are often likelihood or posterior, leading to well-known maximum likelihood and maximum aposteriori inference techniques (Shmulevich et al., 2002; Lähdesmäki et al., 2003). Despite the optimality of these inference techniques, lack of scalability has limited their applications to small GRNs. Several heuristic methods have been developed to scale the inference of Boolean network models; these include scale-free and cluster-based approaches (Hashimoto et al., 2004; Barman and Kwon, 2017), and methods built on evolutionary optimization techniques (Tan et al., 2020; Barman and Kwon, 2018). The former methods aim to build a topology from known seed nodes according to multiple heuristics, whereas the latter ones use evolutionary optimization techniques such as genetic algorithms and particle swarm algorithms for searching over the parameter space. Despite the scalability of these approaches, their incapability to effectively consider the temporal changes in data and efficiently search over possible network models leads to their unreliability in the inference process.

This paper focuses on developing a systematic approach for the inference of GRNs using Boolean network models. Two main challenges in the inference of GRNs are:

- *Large Topology Candidate Space:* The modeling consists of estimating a large number of interacting parameters, which represent the connections between genes that govern their dynamics. This requires searching over a large number of topology candidates and picking the one with the highest likelihood value given the available data. Most existing inference methods for general nonlinear models are developed to deal with continuous parameter spaces, such as maximum likelihood (Johansen et al., 2008; Kantas et al., 2015; Imani and Braga-Neto, 2017; Imani et al., 2020), expectation maximization (Hürzeler and Künsch, 1998; Godsill et al., 2004; Schön et al., 2011; Wills et al., 2013), and multi-fidelity (Imani et al., 2019; Imani and Ghoreishi, 2021) methods. However, these methods cannot be applied for inference over large discrete parameter spaces, such as the large topology candidate space of GRNs. In this paper, we develop a method that is scalable with respect to the number of unknown interactions, and efficiently searches over the large topology candidate space. More specifically, our proposed method enables optimal inference in the presence of a large number of unknown regulations for GRNs with a relatively small number of genes.
- *Expensive Likelihood Evaluation:* The likelihood function, which measures the probability that the available data come from each topology candidate, is often expensive to evaluate. The reasons for that are a large number of genes in GRNs, and the sparsity in the data, which require propagation of the system stochasticity across time and gene states. Given the limitation in the computational resources, evaluation of the likelihood functions for all of the topology candidates is impossible, and one needs to find the topology with the highest likelihood value with a few expensive likelihood evaluations.

This paper derives a scalable topology inference for GRNs observed through temporally sparse data. The proposed framework models the expensive-to-evaluate (log−)likelihood function using a Gaussian Process (GP) regression with a structurally-inspired kernel function. The proposed kernel function exploits the structure of GRNs to efficiently learn the correlation over the topologies, and enables Bayesian prediction of the log-likelihood function for all the topology candidates. Then, a sample-efficient search over topology space is achieved through a Bayesian optimization policy, which sequentially selects topologies for likelihood evaluation according to the posterior distribution of the GP model. The proposed method optimally balances exploration and exploitation, and searches for the global solution without getting trapped in the local solutions. The accuracy and robustness of the proposed framework are demonstrated through comprehensive numerical experiments using a well-known mammalian cell-cycle network.

The remainder of this paper is organized as follows. Section 2 provides a detailed description of the GRN model and the topology inference of GRNs. Further, the proposed topology optimization framework is introduced in Section 3. Section 4 presents various numerical results, and the main conclusions are discussed in Section 5.

## 2 Preliminaries

### 2.1 GRN model

This paper employs a Boolean network with perturbation (BNp) model for capturing the dynamics of GRNs (Shmulevich and Dougherty, 2010; Imani et al., 2018; Hajiramezanali et al., 2019). Previously, several works have successfully employed the BNp model for different purposes such as inference (Dougherty and Qian, 2013; Marshall et al., 2007) and classification (Karbalayghareh et al., 2018). This model properly captures the stochasticity in GRNs, coming from intrinsic uncertainty or unmodeled parts of the systems. Consider a GRN consisting of *d* genes. The *state process* can be expressed as {**X**_*k*_; *k* = 0, 1, …}, where **X**_*k*_ ∈ {0,1}^*d*^ represents the activation/inactivation state of the genes at time *k*. The gene state is updated at each discrete time through the following Boolean signal model:

(1) $$X_{k}=f(X_{k-1})⊕n_{k},$$

for *k* = 1, 2, …, where **n**_*k*_ ∈ {0,1}^*d*^ is Boolean transition noise at time *k*, “⊕” indicates component-wise modulo-2 addition, and **f** represents the *network function*.

**FIGURE 1:**
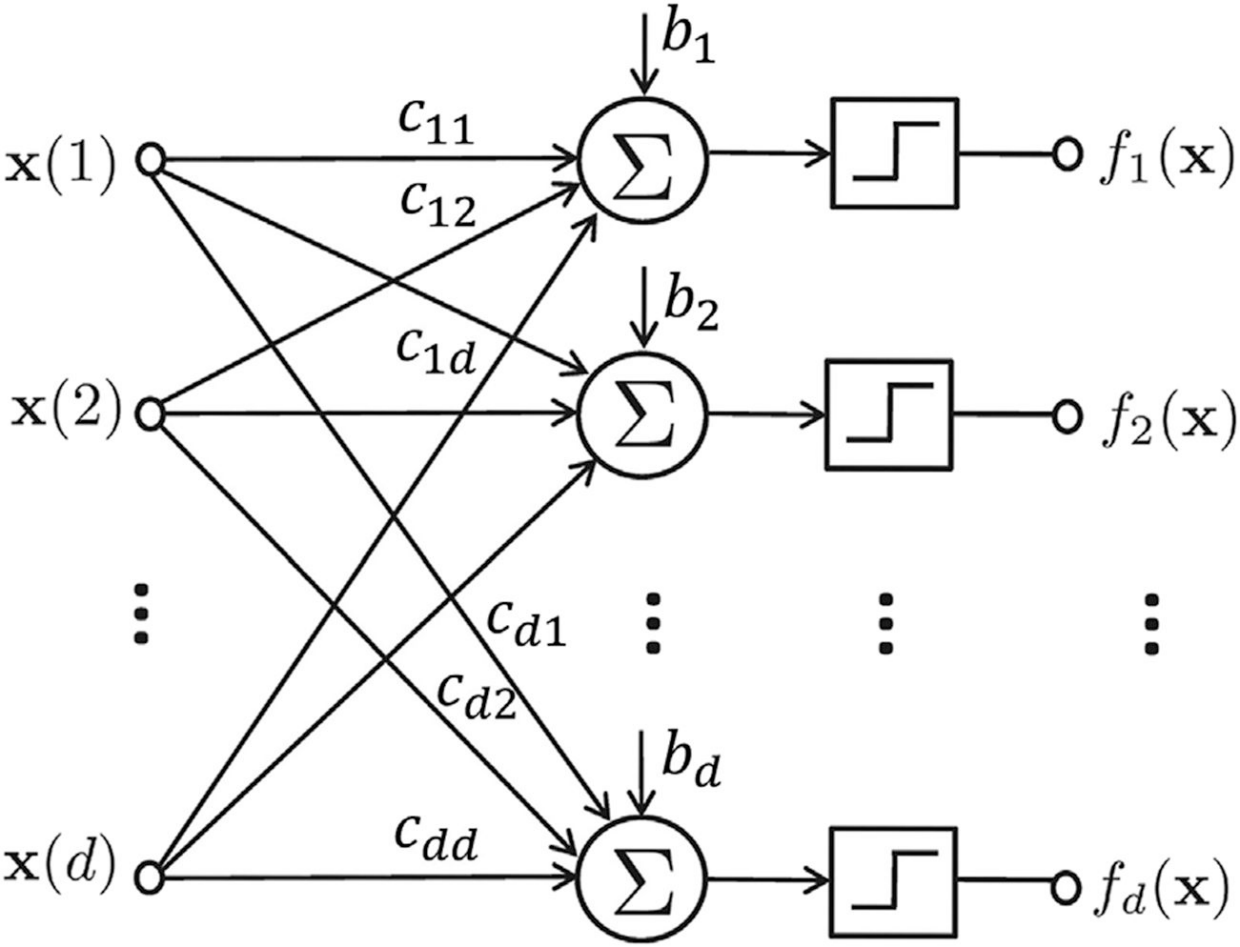
The schematic representation of a regulatory network model. The step functions map outputs to 1 if the input is positive, and 0, otherwise.

The network function in Eq. 1 is expressed in component form as **f** = (*f*_1_, …, *f*_*d*_). Each component *f*_*i*_: {0,1}^*d*^ → {0, 1} is a Boolean function given by:

(2) fi(x)={1,∑j=1dcijx(j)+bi>0,0,∑j=1dcijx(j)+bi≤0,}

for *i* = 1, …, *d*, where *c_ij_* denotes the type of regulation from component *j* to component *i*; it takes +1 and −1 values if there is a positive and negative regulations from component *j* to component *i* respectively, and 0 if component *j* is not an input to component *i*. *b_i_* is a tie-breaking parameter for component *i*; it takes $\frac{+1}{2}$ if an equal number of positive and negative inputs lead to state value +1 and reverse for $\frac{-1}{2}$. The network function in Eq. 2 can also be expressed in matrix form as:

(3) $$f(X_{k-1})=\bar{CX_{k-1}+b},$$

where the threshold operator $\bar{v}$ maps the positive elements of vector **v** to 1 and negative elements to 0, **C** is the connectivity matrix with (**C**)_*ij*_ = *c*_*ij*_ in the ith row and *j*th column, and **b** = [*b*_1_, … *b*_*d*_]^*T*^ represents the bias vector. A schematic representation of the regulatory network model is shown in Figure 1.

In Eq. 1, the noise process **n**_*k*_ indicates the amount of stochasticity in a Boolean state process. For example, **n**_*k*_(*j*) = 1, means that the *j*th gene’s state at time step *k* is flipped and does not follow the Boolean function. Whereas, **n**_*k*_(*j*) = 0 indicates that this state is governed by the network function. We assume that all the **n**_*k*_ components are independent and have a Bernoulli distribution with parameter (*p*), which 0 ≤ *p* < 0.5 refers to the amount of stochasticity in each state variable (i.e., gene).

### 2.2 Topology inference of regulatory networks

In practice, the network function is unknown or partially known, and the unknown parameters need to be inferred through available data. The unknown information is often the elements of the connectivity matrix or bias units. We assume that *L* elements of the connectivity matrix {*c*^1^, …, *c*^*L*^} are unknown. Given that each element takes in values in space { + 1, 0, −1}, there will be 3^*L*^ different possible models (i.e., connectivity matrices) denoted by parameter vectors: Θ = {***θ***^1^, …, ***θ***^3^*L*^^}, where ***θ***^*j*^ = [***θ***^*j*^ (1), …, ***θ***^*j*^(*L*)], and ***θ***^*j*^(*i*) denotes the type of the *i*th unknown interaction/parameter under the *j*th model. It is evident that each parameter vector corresponds to one specific topology/model; therefore, the phrases parameter vector and topology/model are used interchangeably throughout this paper. Further, **C**^***θ**^j^*^ represents the connectivity matrix associated with parameter vector ***θ***^*j*^, while only one parameter vector represents the true underlying system topology. Assuming that *D*_1:*T*_ represents the available data, the inference process can be formulated as:

(4) $$\theta^{⋆}=\underset{\theta \in \Theta }{\text{argmax}}P(D_{1:T}\;|\;\theta ),$$

where *P* (*D*_1:*T*_∣***θ***) is the likelihood function for the topology parameterized by ***θ***. The solution to the optimization problem, θ* in Eq. 4, is known as the maximum likelihood solution. Note that without loss of generality, the proposed method, which will be described in the next section, can be applied to any arbitrary point-based estimator, such as maximum aposteriori.

It should be noted that the unknown parameters could include the bias units in the network model in Eq. 3. Depending on the regulatory network, the bias units are often $\frac{-1}{2}$ for the network in normal conditions, whereas a combination of $\frac{+1}{2}$ and $\frac{-1}{2}$ often represents the network in stress conditions. Therefore, if the network condition is not known, the topology inference could aim to simultaneously estimate the parameters of the connectivity matrix and bias units.

## 3 The proposed framework

### 3.1 Likelihood evaluation

Let {**x**^1^, …, **x**^2^*d*^^} be an arbitrary enumeration of the possible Boolean state vectors (i.e., a GRN with *d* components). The available data in *D*_1:*T*_ can be represented using the vector *I*_1:*T*_ = {*I*_1_, …., *I*_*T*_}, where *I*_*k*_ specifies the index associated with the *k*th state (0 if the state at time step *k* is missing). For instance, $D_{1:6}=\{{\tilde{X}}_{2}=x^{9},{\tilde{X}}_{3}=x^{3},{\tilde{X}}_{6}=x^{11}\}$ contains the information of time steps 2, 3 and 6, and denotes that data at time steps 1, 4 and 5 are missing. In this case, the indicator vector is defined as *I*_1:6_ = {0, 9, 3, 0, 0, 11}.

For any given model ***θ*** ∈ *Θ*, we define the predictive posterior distribution ($\Pi_{k|k-1}^{\theta }$) and posterior distribution ($\Pi_{k|k}^{\theta }$) of the states as:

(5) $$\Pi_{k|k-1}^{\theta }(i)=P\left(X_{k}=x^{i}\;|\;I_{1:k-1},\theta \right),\;\;\Pi_{k|k}^{\theta }(i)=P\left(X_{k}=x^{i}\;|\;I_{1:k},\theta \right),$$

for *i* = 1, …, 2^*d*^ and *k* =1, 2, ….

We define the *transition matrix M^**θ**^* of size 2^*d*^ × 2^*d*^ associated with a GRN model parameterized by ***θ***, through the following notation:

(6) $$(M^{\theta }{)}_{ij}=P\left(X_{r}=x^{i}\;|\;X_{r-1}=x^{j},\theta \right)\;\;=P\left(n_{r}=f^{\theta }\left(x^{j}\right)⊕x^{i}\right)\;\;=p^{\|\bar{C^{\theta }x^{j}+b}⊕x^{i}{\|}_{1}}{\left(1-p\right)}^{d-\|\bar{C^{\theta }x^{j}+b}⊕x^{i}{\|}_{1}},$$

for *i, j* = 1, …, 2^*d*^, where the second and third lines in Eq. 6 are obtained based on the GRN model in Eq. 1.

Let $\Pi_{0|0}^{\theta }(i)=P\;(X_{0}=x^{i}\;|\;\theta )$, for *i* = 1, …, 2^*d*^, be the initial state distribution. If no knowledge about this distribution is available, this can be represented by $\Pi_{0|0}^{\theta }(i)=1∕2^{d}$, for *i* = 1, …, 2^*d*^, and ***θ*** ∈ *Θ*. The predictive posterior distribution can be computed recursively as:

(7) $$\Pi_{k|k-1}^{\theta }=M^{\theta }\Pi_{k-1|k-1}^{\theta }.$$

The posterior probability of states at time step *k* can be computed according to predictive posterior and the available data at time step *k*. If the data at time step *k* is missing, i.e. *I*_*k*_ = 0, the predictive posterior becomes the posterior, as no data is available at time step *k*. This can be written as:

(8) $$\Pi_{k|k}^{\theta }(j)=P\left(X_{k}=x^{j}\;|\;I_{1:k},\theta \right)=P\left(X_{k}=x^{j}\;|\;I_{1:k-1},I_{k}=0,\theta \right)\;\;=\Pi_{k|k-1}^{\theta }(j),\;\text{for}\;j=1,\ldots ,2^{d}.$$

However, if the *i*th state is observed at time step *k*, i.e., *I*_*k*_ = *i*, then the posterior probability of state at time step *k* becomes 1 for state *i*, as full knowledge about **X**_*k*_ = **x**^*i*^ is available. The posterior probability in this case can be expressed as:

(9) $$\;\Pi_{k|k}^{\theta }(i)=P\left(X_{k}=x^{i}\;|\;I_{1:k-1},I_{k}=i,\theta \right)=1,\;\Pi_{k|k}^{\theta }(j)=P\left(X_{k}=x^{j}\;|\;I_{1:k-1},I_{k}=i,\theta \right)=0,\;j\neq i.$$

To summarize, the posterior portability of any state at time *k*, i.e. **X**_*k*_ = **x**^*i*^ can be derived through the following expression:

(10) Πk∣kθ(i)={Πk∣k−1θ(i)ifIk=0,1ifIk=i,0otherwise}

for *i* = 1, …, *2*^*d*^ and *k* = 1, 2, ….

The likelihood value in optimization problem in Eq. 4 can be written in logarithmic format as:

(11) $$L(\theta )≔\log\;P(D_{1:T}\;|\;\theta )=\log\;P(I_{1:T}\;|\;\theta )\;\;=\sum_{k=1}^{T}\log\;P(I_{k}\;|\;I_{1:k-1},\theta ),$$

where

(12) P(Ik∣I1:k−1,θ)={Πk∣k−1θ(Ik)ifIk≠01otherwise}.

The computation of the log-likelihood value for any given topology can be huge due to the large size of the transition matrices with 2^2*d*^ elements. The computational complexity of log-likelihood evaluation is of order *O* (2^2*d*^*T*), where *T* is the time horizon. This substantial computational burden (especially in systems with a large number of components) is the motivation to come up with more efficient ways to solve the problem presented in Eq. 4.

### 3.2 Bayesian optimization for topology optimization

This article proposes a Bayesian optimization approach for scalable topology inference of regulatory networks observed through temporally sparse data. Bayesian optimization (BO) (Frazier, 2018) is a well-known approach that has been extensively used in recent years for optimization problems in domains with expensive to evaluate objective functions. BO has shown great promise in increasing the automation and the quality of the optimization tasks (Shahriari et al., 2016). In this paper, we are dealing with an expensive-to-evaluate likelihood function. A major issue in employing the conventional BO is its ability of dealing with continuous search spaces, whereas the search space in our problem is the topology of regulatory networks, which takes a large combinatorial space. Therefore, some key changes need to be applied to the original BO formulation so that it can be adapted to our problem. The main concepts of this approach are explained in detail in the following paragraphs.

#### 3.2.1 GP Model over the Log-Likelihood Function

The transition matrix (*M*^***θ***^) in Eq. 7 makes the log-likelihood function evaluation in Eqs. 4, 11 computationally expensive, especially when dealing with large scale regulatory networks. Therefore, it is vital to come up with an efficient way of searching over the topology space. In this article, the log-likelihood function *L* (.) is modeled using the Gaussian Process (GP) regression. The GP (Rasmussen and Williams, 2006) is mostly defined over continuous spaces, primarily due to the possibility of defining kernel functions that model the correlation over continuous spaces. In our case, the parameters are discrete interactions (i.e., parameters of the connectivity matrix that take +1, 0, or −1), which prevent constructing the GP model for representing the log-likelihood function over topology space.

This paper takes advantage of the topology structure of GRNs, encoded in connectivity matrix in (3), and defines the following GP model:

(13) L(θ)=GP(μ(θ),k(θ,θ)),

where *μ*(.) shows the mean function, and *k* (.,.) indicates the topology-inspired kernel function. The mean function, *μ*(.), in Eq. 13 represents the prior shape of the log-likelihood function over all the topologies. One possible choice for the mean function is the constant mean function. This mean function carries a single hyperparameter, which can be learned along with the kernel hyperparameters.

Knowing that each parameter vector ***θ*** corresponds to a connectivity matrix **C**^***θ***^, the structurally-inspired kernel function is defined as:

(14) $$k(\theta ,\theta^{\prime })=\sigma_{f}^{2}\;\text{exp}\left(-\frac{\|C^{\theta }-C^{\theta^{\prime }}{\|}^{2}}{l}\right),$$

where ∥**V**∥^2^ is the sum of squares of elements of **V**, 𝒞^***θ***^ and 𝒞^***θ***^′ represent the connectivity matrices related to topologies ***θ*** and ***θ***′ respectively, *l* is the length-scale, and $\sigma_{f}^{2}$ is the scale factor hyperparameters. These hyperparameters quantify how close the topologies are to each other. The more similar two topologies are (i.e., less difference in the connectivity matrices), the more they are correlated, and the kernel function value will be higher for them. While, for more distinct topologies, the kernel will have smaller values.

**FIGURE 2:**
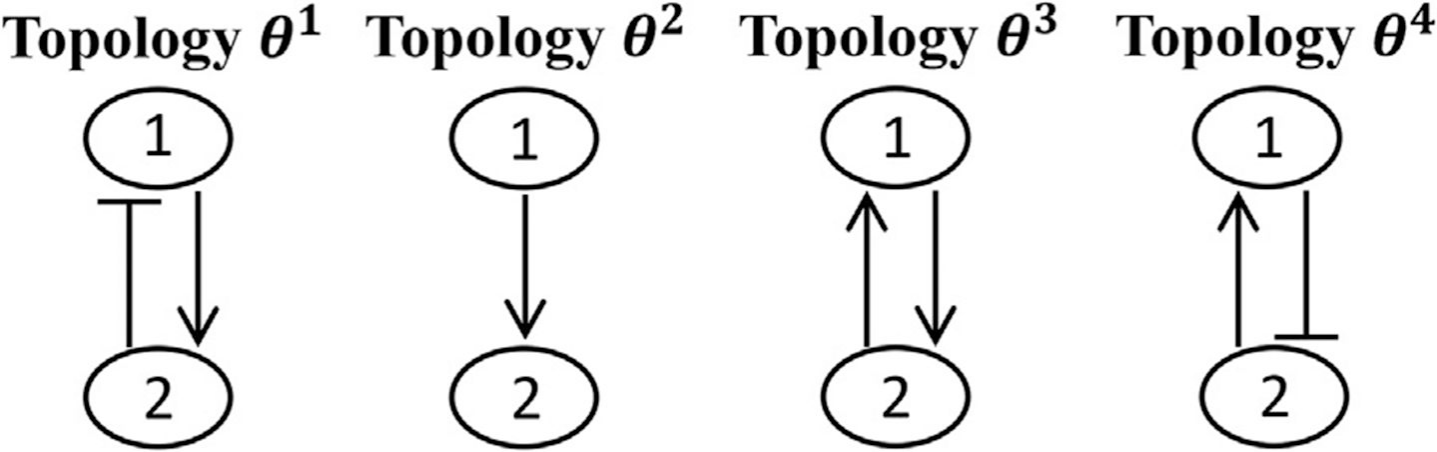
An example of possible models (i.e, topologies) for a GRN with two genes.

Figure 2 represents an example of a few possible topologies for a GRN with two genes. These four possible topologies differ in one or two interactions. If the log-likelihood value for topology ***θ***^1^ is calculated, this information can be used for predicting log-likelihood values for other topologies. The connectivity matrices for these topologies can be expressed as:

(15) $$C^{\theta^{1}}=\left[\begin{array}{cc} 0 & -1\\ 1 & 0 \end{array}\right],C^{\theta^{2}}=\left[\begin{array}{cc} 0 & 0\\ 1 & 0 \end{array}\right],C^{\theta^{3}}=\left[\begin{array}{cc} 0 & 1\\ 1 & 0 \end{array}\right],C^{\theta^{4}}=\left[\begin{array}{cc} 0 & 1\\ -1 & 0 \end{array}\right].$$

The correlation between topology ***θ***^1^ and all the aforementioned topologies, Θ^sub^ = {***θ***^1^, ***θ***^2^, ***θ***^3^, ***θ***^4^} are calculated based on Eq. 14, and expressed through the following kernel vector:

(16) $$K_{\left(\theta^{1},\Theta^{\text{sub}}\right)}=\left[\;k(\theta^{1},\theta^{1})\;k(\theta^{1},\theta^{2})\;k(\theta^{1},\theta^{3})\;k(\theta^{1},\theta^{4})\;\right]\;\;=\left[\sigma_{f}^{2}\;\frac{\sigma_{f}^{2}}{\text{exp}\;(1)}\;\frac{\sigma_{f}^{2}}{\text{exp}\;(4)}\;\frac{\sigma_{f}^{2}}{\text{exp}\;(16)}\right],$$

where the length-scale hyperparameter is assumed to be 1. It can be seen that topology ***θ***^1^ has the maximum correlation with itself, and the correlation rate decreases when we move from topology ***θ***^1^ to ***θ***^4^. This can also be understood in terms of the differences between the interacting parameters, expressed in the connectivity matrices in Eq. 15. Topology ***θ***^2^ is different from ***θ***^1^ in only missing interaction from gene 2 to gene 1. This results in a correlation of $k(\theta^{1},\theta^{2})=\frac{\sigma_{f}^{2}}{\text{exp}(1)}$ between these two topologies. Further, in Eq. 15 we can see that the interaction from gene 2 to gene 1 in model ***θ***^1^ is suppressive (−1), whereas the same interaction is activating (+1) in model ***θ***^3^. This leads to smaller correlation between topologies ***θ***^1^ and ***θ***^3^, $k(\theta^{1},\theta^{3})=\frac{\sigma_{f}^{2}}{\text{exp}(4)}$, in comparison to the correlation between topologies ***θ***^1^ and ***θ***^2^. Finally, in Eq. 15, it can be seen that ***θ***^1^ and ***θ***^4^ have two opposite types of interactions, leading to the $k(\theta^{1},\theta^{4})=\frac{\sigma_{f}^{2}}{\text{exp}(16)}$, which is the smallest correlation between ***θ***^1^ and all the other topologies.

The GP model has the capability of providing the Bayesian representation of the likelihood function across the topology space. Let ***θ***_1:*t*_ = (***θ***_1_, …, ***θ***_*t*_) be the first *t* samples from the parameter space (i.e., samples from the topology candidates) with the associated log-likelihood values *L*_1: *t*_ = [*L*_1_, …, *L*_*t*_]^*T*^ (i.e., *L*_1_ = *L* (***θ***_1_) in Eq. 11). The posterior distribution of ℒ(***θ***) in Eq. 13 is derived as:

(17) $$L(\theta )\;|\;\theta_{1:t},L_{1:t}∼N\left(\mu_{\theta }^{t},\Sigma_{\theta }^{t}\right),$$

where $\mu_{\theta }^{t}$ and $\Sigma_{\theta }^{t}$ are the mean and variance for a specific model ***θ*** ∈ *Θ* respectively. These values can be obtained as:

(18) $$\mu_{\theta }^{t}=\mu (\theta )+K_{\left(\theta ,\theta_{1:t}\right)}K_{\left(\theta_{1:t},\theta_{1:t}\right)}^{-1}\left(L_{1:t}-\mu (\theta_{1:t})\right),\;\Sigma_{\theta }^{t}=k_{\left(\theta ,\theta \right)}-K_{\left(\theta ,\theta_{1:t}\right)}K_{\left(\theta_{1:t},\theta_{1:t}\right)}^{-1}K_{\left(\theta ,\theta_{1:t}\right)}^{T},$$

where ***μ***(***θ***_1: *t*_) = *μ*(***θ***_1_), …, *μ*(***θ***_*t*_)]^*T*^, and

(19) $$K_{\left(\Theta ,\Theta^{\prime }\right)}=\left[\begin{array}{ccc} k(\theta_{1},\theta_{1}^{\prime }) & \ldots & k(\theta_{1},\theta_{r}^{\prime })\\ \vdots & \ddots & \vdots \\ k(\theta_{l},\theta_{1}^{\prime }) & \ldots & k(\theta_{l},\theta_{r}^{\prime }) \end{array}\right],$$

for **Θ** = {***θ***_1_, …, ***θ***_*l*_}, $\Theta^{\prime }=\{\theta_{1}^{\prime },\ldots ,\theta_{r}^{\prime }\}$. Using the aforementioned formulation, the GP constructs the log-likelihood function as a zero-mean Bayesian surrogate model with covariance *k* (.,.). Further, at iteration *t*, the log-likelihood function can be computed by employing the already chosen and evaluated log-likelihood values for topologies ***θ***_1:*t*_, i.e., *L*_1:*t*_. The uncertainty of the surrogate model will be reduced as we evaluate the likelihood function for more topologies.

The GP hyperparameters, which consist of the hyperparameters of the topology-inspired kernel function and the mean function, can be learned by optimizing the marginal likelihood function of the GP model at each iteration through:

(20) $$(L_{1:t}\;|\;\theta_{1:t})∼N\left(\mu (\theta_{1:t}),K_{\left(\theta_{1:t},\theta_{1:t}\right)}\right).$$

#### 3.2.2 Sequential Topology Optimization

The notion of efficient topology optimization is to come up with an efficient way of searching over all the topology space so that we utilize a minimum number of computationally expensive likelihood evaluations and eventually find the optimal topology, which yields the largest likelihood value.

As mentioned in Section 3.1, evaluation of the log-likelihood function for each topology is a computationally expensive task. Therefore, in here the sample-efficient and sequential topology selection is achieved as:

(21) $$\theta_{t+1}=\underset{\theta \in \Theta }{\text{argmax}}\alpha_{t}\;(\theta ),$$

where *α_t_*(***θ***) represents the *acquisition function* in the Bayesian optimization context, which is determined over the GP model posterior at iteration *t*. Multiple acquisition functions exist in the context of Bayesian optimization. For instance, *probability improvement* (Shahriari et al., 2016) is one of the most traditional acquisition functions, which makes selections to increase the likelihood of improvement in each iteration of BO. Other examples for acquisition functions include *expected improvement* (Mockus et al., 1978; Jones et al., 1998; Brochu et al., 2010), *upper confidence bound* (Auer, 2003), *knowledge gradient* (Wu et al., 2017; Frazier, 2009), and *predictive entropy search* (Henrández-Lobato et al., 2014). In this work, we use the expected improvement acquisition function, which is the most commonly used acquisition function. This acquisition function balances the exploration and exploitation trade-off, and furthermore has a closed form solution. The expected improvement acquisition function is defined as (Mockus et al., 1978; Jones et al., 1998):

(22) $$\alpha_{t}\;(\theta )=\left(\mu_{\theta }^{t}-L_{\max}^{t}\right)\Phi \left((\mu_{\theta }^{t}-L_{\max}^{t})/\sqrt{\Sigma_{\theta }^{t}}\right)\;\;+\sqrt{\Sigma_{\theta }^{t}}\phi \left((\mu_{\theta }^{t}-L_{\max}^{t})/\sqrt{\Sigma_{\theta }^{t}}\right),$$

Where *ϕ*(.) and Φ(.) refer to the probability density function and cumulative density function of standard normal distribution, $L_{\max}^{t}=\max\;\{L_{1},\ldots L_{t}\}$ is the maximum log-likelihood value until the latest turn, and $\mu_{\theta }^{t}$ and $\Sigma_{\theta }^{t}$ are the mean and variance of the GP model at iteration *t* as defined in Eq. 18.

The acquisition function in Eq. 22 holds a closed-form solution and requires the mean and variance of the GP model for any given topology. To solve Eq. 21 for large regulatory networks with a large number of unknown interactions, we can implement some heuristic optimization methods including particle swarm optimization technique (Kennedy and Eberhart, 1995), genetic algorithm (Anderson and Ferris, 1994; Whitley, 1994), or the breadth-first local search (BFLS) (Atabakhsh, 1991) to obtain the model with the largest acquisition value. After the model with maximum acquisition value (***θ***_*t*+1_) is chosen, the next log-likelihood evaluation is carried for topology ***θ***_*t*+1_ to derive the log-likelihood value *L*_*t*+1_. The GP model is then updated based on all the new information, defined as ***θ***_1:*t*+1_ = (***θ***_1:*t*_, ***θ***_*t*+1_) and *L*_1: *t*+1_ = [*L*_1: *t*_, *L*_*t*+1_]^*T*^.

The proposed Bayesian topology optimization continues its sequential process over all the topology space of the regulatory networks for a fixed number of turns, or until no significant change in the maximum log-likelihood value in consecutive iterations is spotted. When the optimization ends, the topology with the largest evaluated likelihood value is selected as the system topology, meaning that:

(23) $$\theta^{⋆}≔\theta_{i^{⋆}},\;\text{where}\;i^{⋆}=\underset{i=1,\ldots ,t+1}{\text{argmax}}L_{i}.$$

**FIGURE 3:**
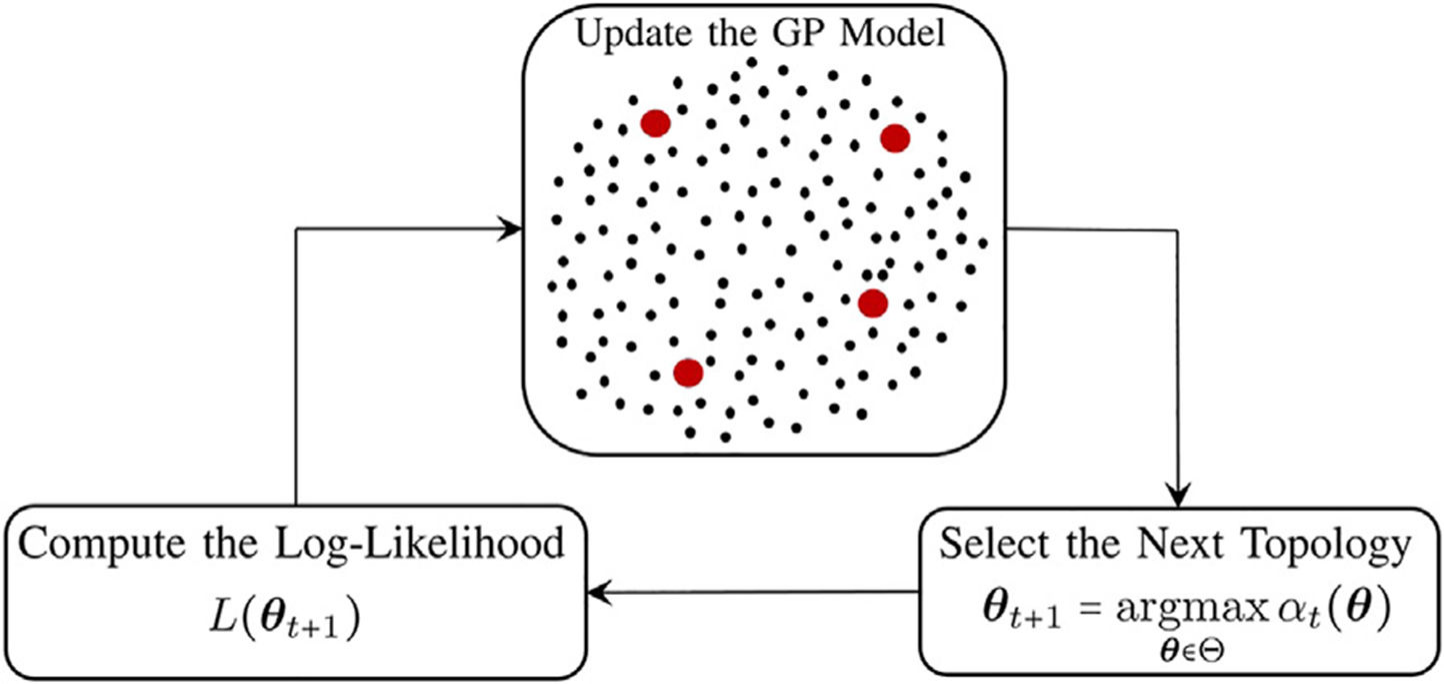
Schematic diagram of the proposed topology inference in GRNs.

The inference process consists of three main components. Figure 3 represents the schematic diagram of the proposed method. The GP model predicts the log-likelihood values over the possible topology candidates, denoted by the black dots in Figure 3. The red dots denote the evaluated log-likelihood values for the selected topologies up to the current iteration. Using the posterior distribution of the GP model, the next topology with the highest acquisition function is selected, followed by the log-likelihood evaluation for the selected topology. The GP is then updated based on the selected topology and the evaluated log-likelihood, and this sequential process continues until a stopping criterion is met.

The detailed steps of the proposed inference method are described in Algorithm 1. **Θ** denotes the topology space, and *D*_1:*T*_ represents the available data. Line 3 to line 8 of the algorithm creates the state index associated with the data *D*_1:*T*_. The sequential topology optimization process is then carried out from line 10 to line 19, where in each loop, the log-likelihood value for a topology selected by the proposed Bayesian optimization technique is computed, followed by the GP posterior update and the next topology selection. Finally, upon the termination of the inference process, the topology with the maximum log-likelihood is chosen in line 20 as the inferred topology. The computation of the log-likelihood determines the complexity of the algorithm at each step of our proposed method, which is of order *O* (2^2*d*^*T*). This means that the complexity at each step of the proposed method is the same as one log-likelihood evaluation. The log-likelihood evaluation at each iteration is used to update our knowledge (posterior), and to help choose the best candidate for future iterations.

**Table:** 

Algorithm 1. The Proposed method for inference of regulatory networks through temporally-sparse data.
1:Topology spaceΘ;dataD1:T;initialize the hyperparameters of the Gaussian processGP(μ(.),k(.,.)),t=0.2:Arbitrary enumeration of the possible Boolean state vectors:X=[x1,…,x2d]3:Initialization:I=0→1×T4:fork=1,2,…,Tdo5:fori=1,…,2ddo6:Ik={iifDkis not missing andDk=Xi0ifDkis missing}7:endfor8:endfor9:repeatSequentialTopologySelection¯10:Pick the topology with maximum acquisition value:θt+1=argmaxθ∈Θαt(θ)−Eq. 21Log−LikelihoodComputation¯11:Initialization:Π0∣0θt+1(i)=P(X0=xi∣θt+1),fori=1,…,2d,Lt+1=0.12:fork=1,2,…,Tdo13:Predictive Posterior Distribution:Πk∣k−1θt+1=Mθt+1Πk−1∣k−1θt+1.14:Posterior Distribution:Πk∣kθt+1(i)={Πk∣k−1θt+1(i)ifIk=0,1ifi=Ik,0otherwise}15:Log−Likelihood Update:Lt+1=Lt+1+logP(Ik∣I1:k−1,θt+1),whereP(Ik∣I1:k−1,θt+1)={Πk∣k−1θt+1(Ik)ifIk≠01otherwise}16:endforUpdatetheGPModel¯17:Update the hyperparameters of the GP according to(θ1:t+1,L1:t+1).18:t=t+1.19:untilthe stopping criterion is met20:The Inferred Topology:θ∗≔θi∗,wherei∗=argmaxi=1,…,tLi.

## 4 Numerical experiments

**FIGURE 4:**
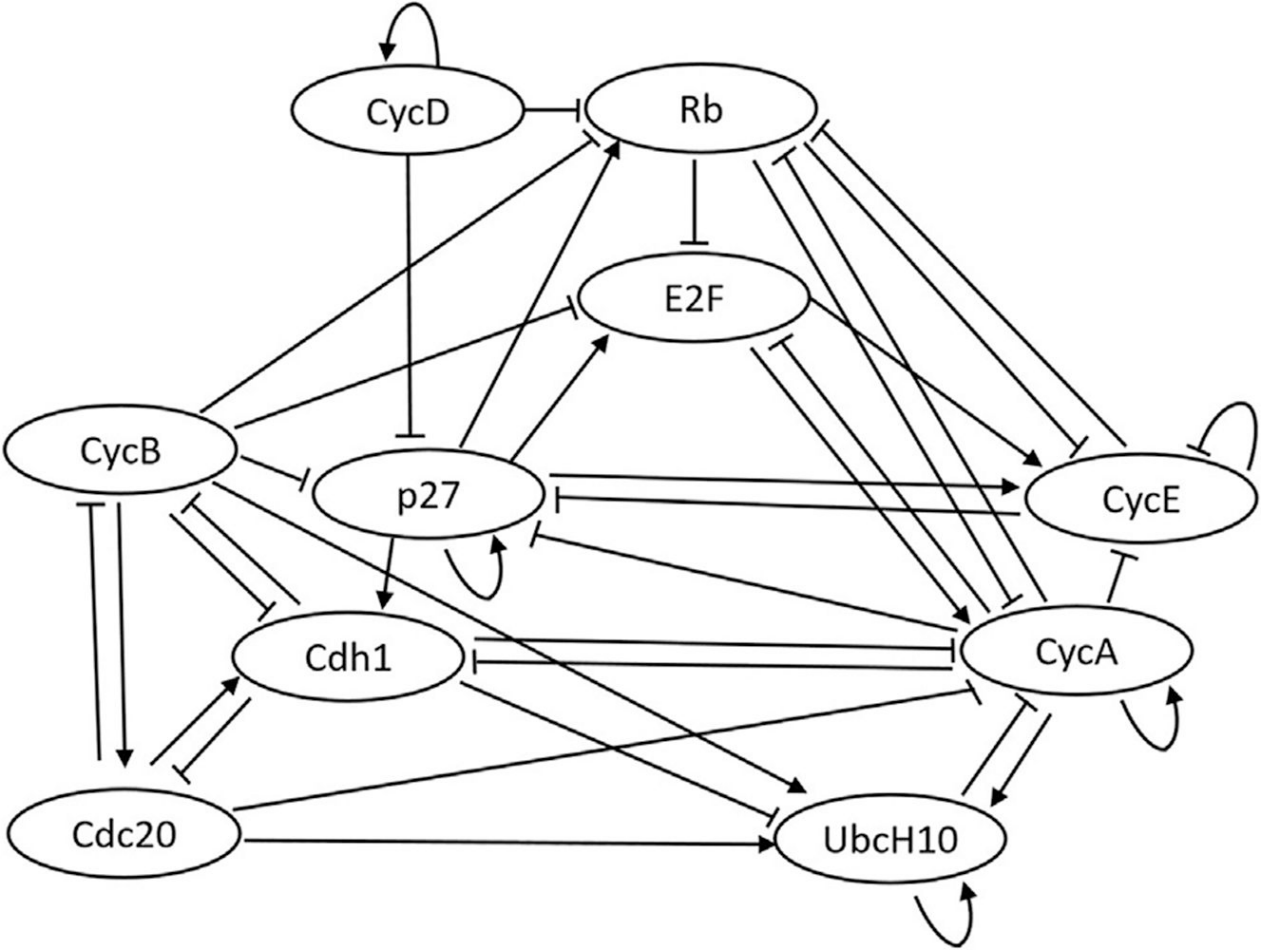
Pathway diagram for the cell-cycle network.

The code repository for replicating the numerical experiments of this paper is included in the data availability statement at the end of this paper. The well-known mammalian cell-cycle network (Fauré et al., 2006) is used to evaluate the performance of our proposed method. Figure 4 presents the pathway diagram of this network. The state vector for this network is assumed as the following **x** =(CycD, Rb, p27, E2F, CycE, CycA, Cdc20, Cdh1, UbcH10, CycB). The division of mammalian cells depends on the overall organism growth, controlled using signals that activate cyclin D (CycD) in the cell. As can be seen in the state vector, the mammalian cell-cycle network contains 10 genes (*d* = 10). The settings used for our experiments are as follows: data length of 100 (*k* = 100), process noise of 0.1 (*p* = 0.1), and missing data percentage (sparsity) of 50%. Furthermore, 10 regulations of the connectivity matrix are assumed to be unknown (*L* = 10), and a maximum of 100 likelihood evaluations are used for the inference process. All the parameters used throughout the numerical experiments are expressed in Table 1.

The connectivity matrix and bias vector in Eq. 3 for the mammalian cell-cycle network can be written as:

(24) $$C=\left[\begin{array}{cccccccccc} +1 & 0 & 0 & 0 & 0 & 0 & 0 & 0 & 0 & 0\\ -1 & 0 & +1 & 0 & -1 & -1 & 0 & 0 & 0 & -1\\ -1 & 0 & +1 & 0 & -1 & -1 & 0 & 0 & 0 & -1\\ 0 & -1 & +1 & 0 & 0 & -1 & 0 & 0 & 0 & -1\\ 0 & -1 & +1 & +1 & -1 & -1 & 0 & 0 & 0 & 0\\ 0 & -1 & 0 & +1 & 0 & +1 & -1 & -1 & -1 & 0\\ 0 & 0 & 0 & 0 & 0 & 0 & -1 & 0 & 0 & +1\\ 0 & 0 & +1 & 0 & 0 & -1 & +1 & 0 & 0 & -1\\ 0 & 0 & 0 & 0 & 0 & +1 & +1 & -1 & +1 & +1\\ 0 & 0 & 0 & 0 & 0 & 0 & -1 & -1 & 0 & 0 \end{array}\right],\;b={\left[\frac{-1}{2}\;\frac{-1}{2}\;\frac{-1}{2}\;\frac{-1}{2}\;\frac{-1}{2}\;\frac{-1}{2}\;\frac{-1}{2}\;\frac{-1}{2}\;\frac{-1}{2}\;\frac{-1}{2}\right]}^{T}.$$

In this section, we are assuming that the connectivity matrix is not fully known. This network has 10 genes, and there is a total of 2^10^ = 1, 024 possible states for this network. Consequently, the transition matrix size is 2^10^ × 2^10^, which causes the likelihood evaluation to be computationally expensive for any possible topology. Using our proposed method, we show that the optimal topology with the largest log-likelihood value can be inferred with few likelihood evaluations; hence, we offer an efficient search over all possible topologies.

In all of the experiments, 10 unknown interactions (*c_ij_*) were considered. Each of the unknown interactions can take their values in the set { + 1, 0, −1}, which leads to 3^10^ = 59,049 different possible system models, i.e., Θ = {***θ***^1^, …, ***θ***^3^10^^}. The 10 randomly chosen unknown regulations, which are elements of the connectivity matrix in Eq. 24, are:

(25) $$c_{21}=-1,\;\;c_{35}=-1,\;\;c_{3\;10}=-1,\;\;c_{42}=-1,\;\;c_{54}=+1\;c_{67}=-1,\;\;c_{69}=-1,\;\;c_{83}=+1,\;\;c_{96}=+1,\;\;c_{98}=-1.$$

We also considered a uniform prior distribution for the initial states, i.e., $\Pi_{0|0}^{\theta }(i)=\frac{1}{2^{10}}$ for all ***θ*** ∈ *Θ* and *i* = 1, 2, …, 2^10^. Furthermore, all the experiments are repeated for 10 independent runs, and the average results along the confidence bounds are reported in all the figures. Note that the randomness of early results come from the process noise (*p*), and the way the sequential topology optimization is being performed in each run.

**FIGURE 5:**
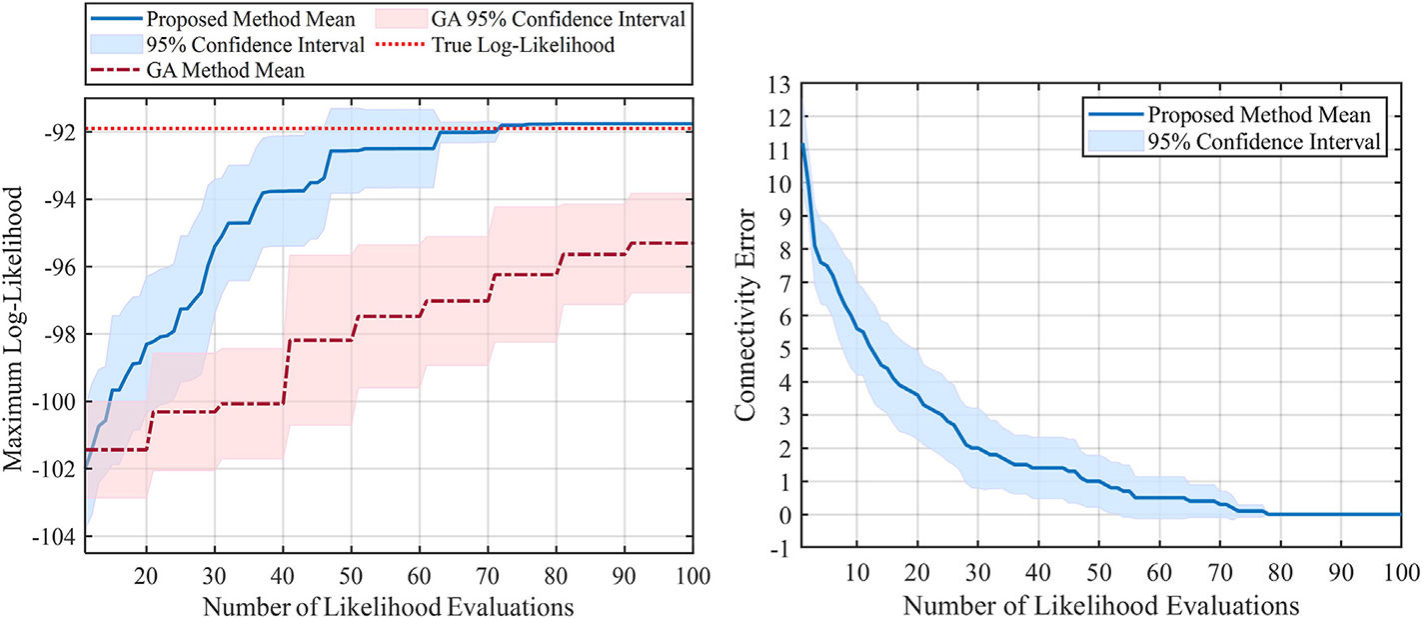
Results of the mammalian cell-cycle network with 10 unknown interactions.

For the first set of experiments, the performance of the proposed method is shown using two plots in Figure 5. The left plot represents the progress of the log-likelihood value of the inferred model with respect to the number of likelihood evaluations, meaning that it shows the maximum log-likelihood value obtained during the optimization process. Larger log-likelihood values mean that the chosen model can better represent the true model (i.e., the available data is more likely to come from models with larger likelihood values). As a comparison, we also repeated the same experiment using Genetic Algorithm (GA) (Anderson and Ferris, 1994; Whitley, 1994), which is a powerful and well-known solver for non-continuous problems. By looking at the left plot in Figure 5, we can see that the inference by the proposed method, indicated by the solid blue line, is better than the GA method (dashed red line). This superiority can be seen in terms of the mean and confidence intervals in Figure 5. As we evaluate more likelihoods for different models, the likelihood of the proposed method’s inferred model gets closer to the optimal log-likelihood value, indicated by the dotted red line. Hence, our proposed method is capable of reaching a better log-likelihood with less number of likelihood evaluations and has a more efficient way of searching over all the possible models. Furthermore, the 95% confidence interval is illustrated in the same plot for both methods during this experiment. We can observe that the proposed method’s confidence interval keeps getting smaller, and roughly after 70 evaluations, the confidence interval tends to go zero. This indicates the robustness of the proposed method, where after roughly 70 iterations, the log-likelihood gets to its optimal value at different independent runs. By contrast, the results from the GA still show a large confidence interval even after 100 evaluations, and its average is far less than the optimal log-likelihood value.

The right plot of Figure 5 shows the progress of the connectivity error during the optimization process (i.e., number of likelihood evaluations) obtained by the proposed method. Let C* be the vectorized true connectivity matrix indicated in Eq. 24, and **C**^*t*^ be the vectorized inferred connectivity matrix at *t*th likelihood evaluation. The connectivity error at iteration *t* is defined as ∥C* − **C**^*t*^∥_1_. Evidently, we will have a better estimate of the true model as this error gets closer to zero. In the right plot, we can see that the connectivity error decreases as we do more evaluations, and after about 75 likelihood evaluations, the error gets to zero, meaning that we successfully inferred the true connectivity matrix. Also, as expected, we can see that the 95% confidence interval gets smaller as we do more evaluations and eventually gets close to zero after about 75 evaluations.

**FIGURE 6:**
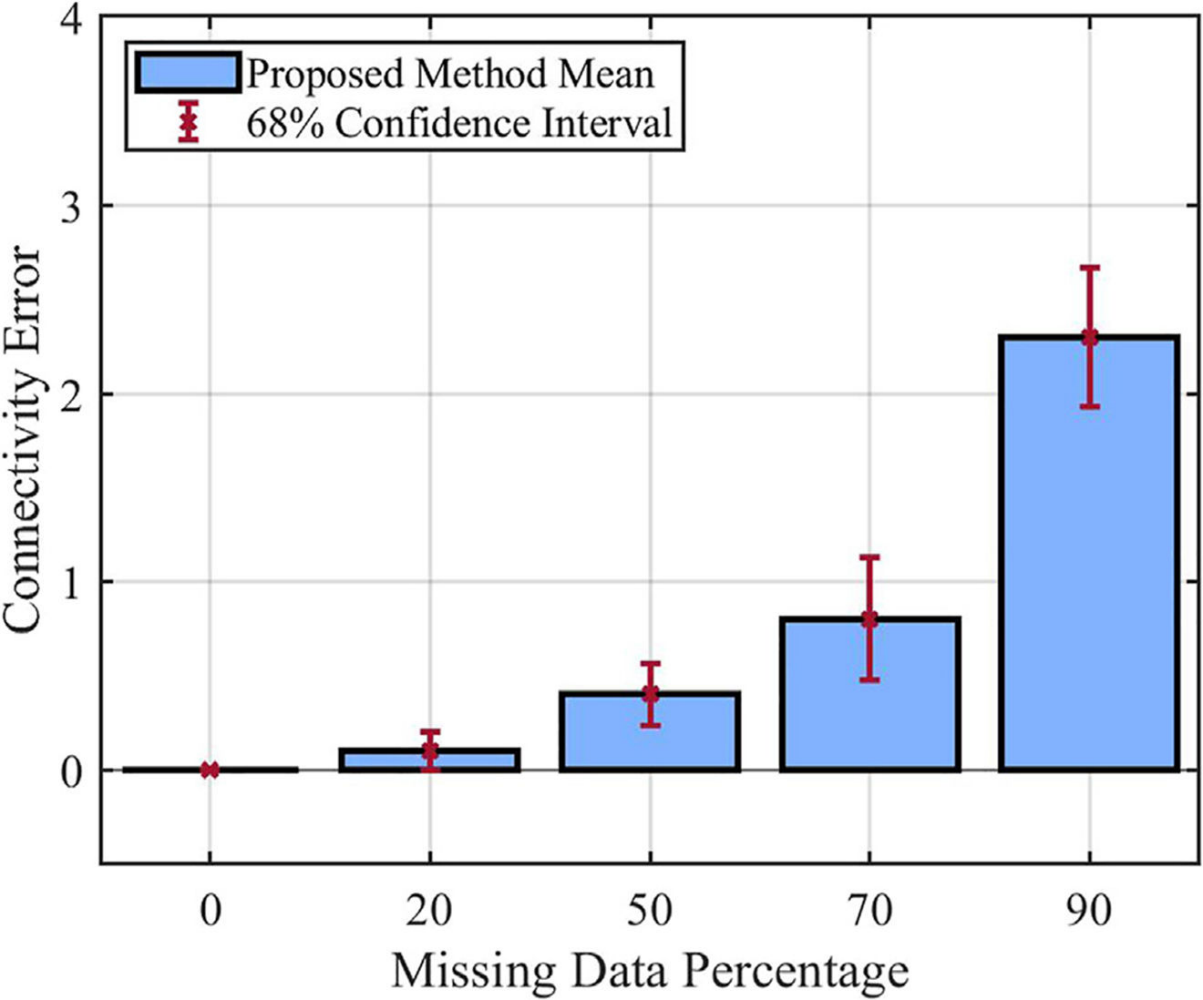
Performance of the proposed method with respect to percentage of missing data.

In the second set of experiments, we aim to investigate the effect of missing data percentage on the performance of the proposed method. It is expected that with more missing data, it would be more difficult to infer the relationship between different components of the system; hence the connectivity error for the inferred model would be larger. For these experiments, we changed the missing data percentage from 0% to 90% and used Bernoulli noise value 0.2. Other parameters are fixed based on Table 1. The mean of the inferred models’ connectivity error obtained from these experiments, along with their 68% confidence interval are presented as bar plots in Figure 6. As expected, these results demonstrate that the mean of connectivity error increases as the missing data percentage gets larger.

**FIGURE 7:**
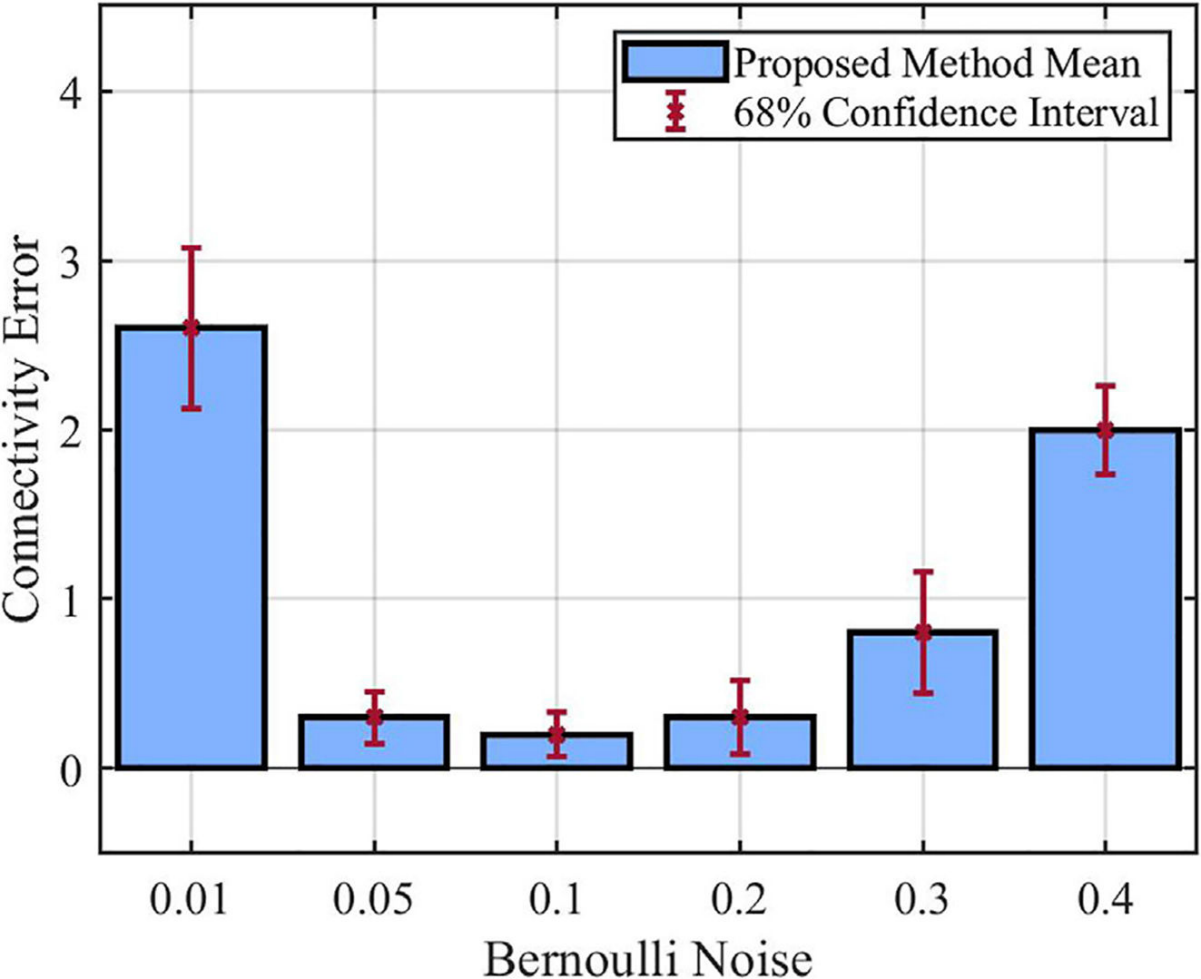
Performance of the proposed method in presence of different Bernoulli noise.

The final set of experiments focuses on how the Bernoulli noise affects the performance of the proposed method. In all these experiments, we consider 50% missing data percentage, and we change the Bernoulli noise from 0.01 to 0.4. For performance comparison, the mean of the inferred models’ connectivity error derived from these experiments is shown using bar plots in Figure 7. In this bar plot, we can observe that the connectivity error is large for the Bernoulli noise of 0.01. As the noise increases to 0.05 and 0.1, the connectivity error keeps decreasing. However, increasing the noise to 0.2, 0.3, and finally 0.4 results in a continuous increase in the connectivity error. These results demonstrate the relationship between network stochasticity and data informativity needed for the inference process. For a small process noise (*p* = 0.01), the network is typically trapped in attractor states, which precludes the observation of the entire state space. This leads to the issue of statistical non-identifiability, which refers to the situation where multiple models are not clearly distinguishable using the available data. Once the noise value is slightly increased (*p* = 0.05, *p* = 0.1), the network gets out of its attractor states more often, which enhances the performance of the inference process. Finally, for too large process noise values (*p* = 0.2, *p* = 0.3, *p* = 0.4), the state transitions become more chaotic, making it more difficult to infer the true relations between the components.

## 5 Conclusion

This paper presents a highly scalable topology inference method for gene regulatory networks (GRNs) observed through temporally sparse data. The Boolean network model is used for capturing the dynamics of the GRNs. The inference process consists of inferring the interactions between genes or equivalently selecting a topology for the system among all the possible topologies that have the highest likelihood value. Evaluating the likelihood function for any given topology is expensive, preventing exhaustive search over the large possible topology space. The proposed method models the log-likelihood function by a Gaussian Process (GP) model with a structurally-inspired kernel function. This GP model captures the correlation between different possible topologies and provides the Bayesian representation of the log-likelihood function. Using the posterior distribution of the GP model, Bayesian optimization is used to efficiently search over the topology space.

The high performance of our proposed method is shown using multiple experiments on the well-known mammalian cell-cycle network. We have also repeated all the experiments multiple times to obtain a confidence interval and further demonstrate the accuracy and robustness of the solutions obtained by our method. In the first experiment, we considered the topology inference of the mammalian cell-cycle network with 10 unknown interactions and 50% missing data. From comparing the results of topology inference using our proposed method and genetic algorithm, we observed that our method is more efficient in searching over the topology space and reaches an optimal model with fewer likelihood evaluations. Meanwhile, the small confidence interval of our method justified the robustness of the solutions. The second experiment investigated the effect of missing data on the performance of the proposed inference method. From the results, we understand that as expected, with more missing data, the method’s accuracy reduces, and the inference error becomes larger. Finally, in the third experiment, we studied the performance of our method in the presence of different Bernoulli noise (i.e., stochasticity in the state process). The results show that for small stochasticity, the accuracy of the inference is low, as the system spends most of its time in a few states (i.e., attractors) and the interactions between different components of the system are not distinguishable. As the stochasticity increases, the accuracy of the proposed method increases (as the error decreases) until a certain point, and after that again, the accuracy starts decreasing. This is because too much stochasticity turns the system into a more chaotic form, making the inference of the true model more challenging.

## Figures and Tables

**TABLE 1:** Parameter values of mammalian cell-cycle network experiments.

Parameter	Value
Trajectory Length, *k*	100
Number of Likelihood Evaluations	100
Number of Genes, *d*	10
Number of Unknown Regulations, *L*	10
Process Noise, *p*	0.1
Missing Data Percentage	50%

## Data Availability

The datasets presented in this study can be found in online repositories. The names of the repository/repositories and accession number(s) can be found below: https://github.com/ImaniLab/Frontiers-2022.
